# Sperm Cell Population Dynamics in Ram Semen during the Cryopreservation Process

**DOI:** 10.1371/journal.pone.0059189

**Published:** 2013-03-27

**Authors:** Manuel Ramón, M. Dolores Pérez-Guzmán, Pilar Jiménez-Rabadán, Milagros C. Esteso, Olga García-Álvarez, Alejandro Maroto-Morales, Luis Anel-López, Ana J. Soler, M. Rocío Fernández-Santos, J. Julián Garde

**Affiliations:** 1 CERSYRA, Junta de Comunidades de Castilla-La Mancha, Valdepeñas, Spain; 2 Dpto. Reproducción Animal, INIA, Madrid, Spain; 3 SaBio (CSIC-UCLM-JCCM), Campus Universitario s.n., Albacete, Spain; University Hospital of Münster, Germany

## Abstract

**Background:**

Sperm cryopreservation has become an indispensable tool in biology. Initially, studies were aimed towards the development of efficient freezing protocols in different species that would allow for an efficient storage of semen samples for long periods of time, ensuring its viability. Nowadays, it is widely known that an important individual component exists in the cryoresistance of semen, and efforts are aimed at identifying those sperm characteristics that may allow us to predict this cryoresistance. This knowledge would lead, ultimately, to the design of optimized freezing protocols for the sperm characteristics of each male.

**Methodology/Principal Findings:**

We have evaluated the changes that occur in the sperm head dimensions throughout the cryopreservation process. We have found three different patterns of response, each of one related to a different sperm quality at thawing. We have been able to characterize males based on these patterns. For each male, its pattern remained constant among different ejaculates. This latter would imply that males always respond in the same way to freezing, giving even more importance to this sperm feature.

**Conclusions/Significance:**

Changes in the sperm head during cryopreservation process have resulted useful to identify the ability of semen of males for freezing. We suggest that analyses of these response patterns would represent an important tool to characterize the cryoresistance of males when implemented within breeding programs. We also propose follow-up experiments to examine the outcomes of the use of different freezing protocols depending on the pattern of response of males.

## Introduction

Understanding the switching of phenotypes in response to environmental changes is at the forefront of biological research in diverse areas of study [1]. One of these areas is focused on the study of cell response to low temperatures. Cell cryopreservation has become an indispensable tool in biology. Biological materials can be safely kept and used after a very long period of time. For the particular case of spermatozoa, cryopreservation is used in livestock management and in the conservation of wild and domestic species, as a complementary tool for managing live animals and preserving their genetic diversity.

Several studies have been conducted to understand the changes that occur during cryopreservation of spermatozoa and develop a protocol to ensure a successful storage (see [2] for references and general discussion). These studies have focused on different traits such as sperm motility and morphometry, integrity of sperm membranes or DNA status among others [3]–[8]. As a result of these studies, standard cryopreservation protocols have been developed for many species, both domestic and wild.

One important problem for standardizing sperm cryopreservation protocols is that spermatozoa from different individuals exhibit significant different responses to the same freezing treatment [2], [9]–[11]. Males can be classified into “bad” and “good” freezers depending on the ability of their spermatozoa to resist to the cryopreservation process. Many studies have been conducted to identify those characteristics that favour the freezability of spermatozoa [3], [12]–[13]. The ultimate goal of these studies is twofold: on the one hand, to be able to know in advance the sperm cryoability of a male, and on the other hand, the design of individualized cryopreservation protocols aimed at maximizing the performance of this technique. Both goals support the idea that sperm cryosurvival can be under genetic control [14].

In this study, the use of Manchega sheep breed has been motivated by low fertility rates derived from the use of frozen semen in artificial insemination in sheep, compared to those obtained from the use of cooled semen. Benefits resulting from the use of frozen semen are clear. However, the poor ability of ram spermatozoa to resist the freezing-thawing process [15]–[16] and, therefore, the low performance derived from its use are responsible of the low dissemination of this technique in the breeding programs of numerous sheep breeds, as is the case of the Manchega sheep breed. This fact together with the individual variability above-mentioned has resulted in a growing interest in the characterization of the sperm cryoability of a male and the development of individual-specific protocols of sperm cryopreservation.

Here we present a novel approach to characterize the sperm cryoresistence of a male. We use morphometry of sperm heads to monitor changes that occur during cryopreservation. As other authors, we examine spermatozoa before and after cryopreservation, but instead of considering these stages in a static way and perform comparison among them, we propose to consider them all together and define patterns of response. In this study, males were first assigned to a group based on the changes that occur on head morphometry of spermatozoa during cryopreservation. Then, we examined the differences among these groups and hypothesize on the causes of this differential response to cryopreservation.

## Results

Among the sperm head parameters measured, only those that better characterize the three ejaculate groups were considered in the results.

**Figure 1:**
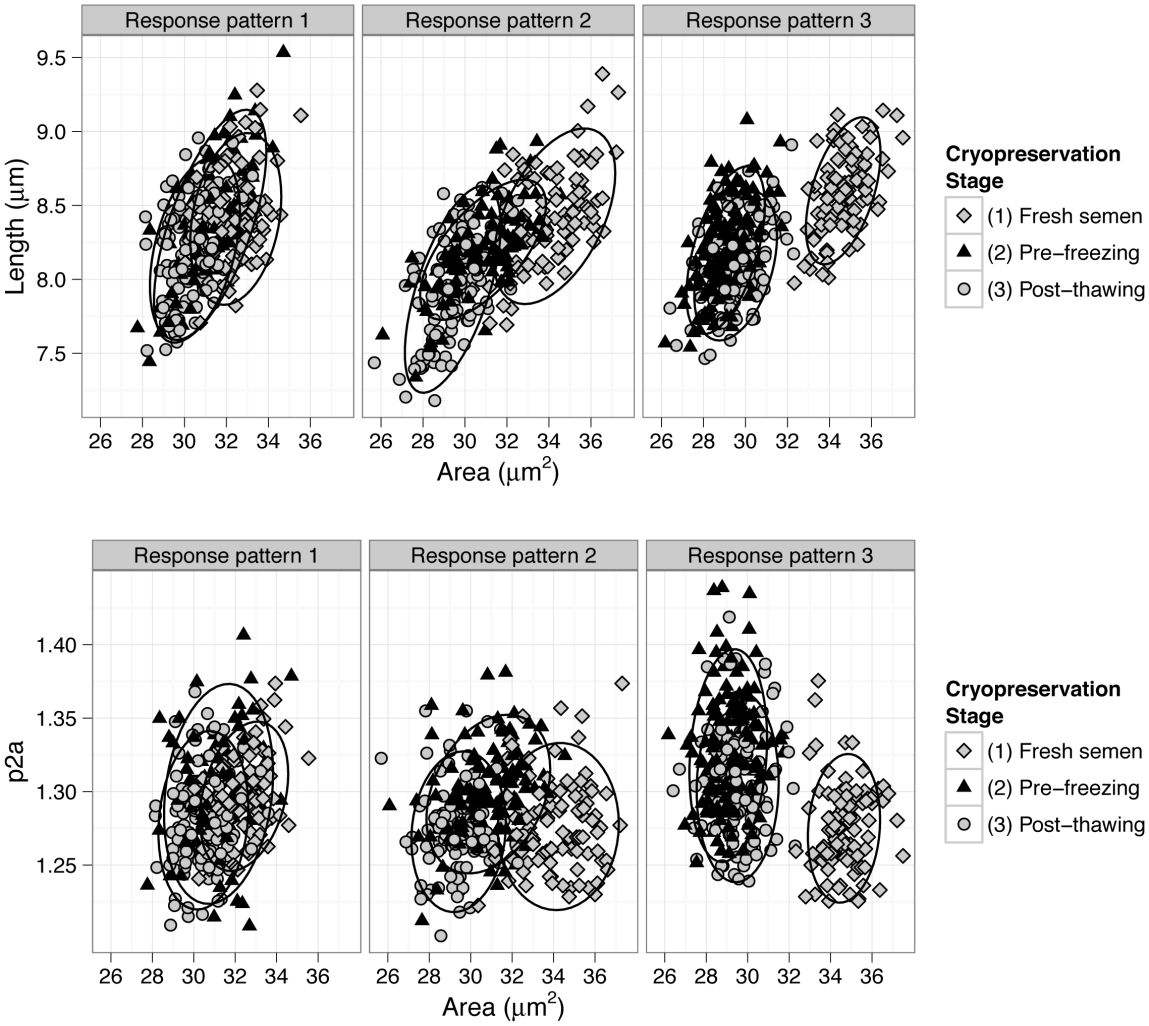
Patterns of response observed in the sperm population during the cryopreservation process. Data plotted correspond to three males, each one showing a different response pattern. *p2a*: perimeter to area (Perimeter^2^/4·π·Area).

In this study, three different patterns of response were observed on males throughout the cryopreservation process (Figure 1). From the 38 males used in this study, 44.7% showed the response pattern 1, 26.3% showed the response pattern 2, and 28.9% showed the response pattern 3. Sperm head dimensions among ejaculates were quite similar for each male with coefficients of variation within a male (between ejaculates) lower than 10 (ranging from 1.14 to 9.30) for all the sperm head parameters. Males always showed the same pattern of response to cryopreservation regardless of the ejaculate.

The males grouped under the ***“response pattern 1”*** showed no significant changes in their sperm head dimensions during cryopreservation (Table 1). Changes on sperm dimension were close to 0 and quite similar between stages of the crypreservation process.

**Table 1:** Head morphomertic characteristics of spermatozoa during the cryopreservation process for the three patterns of response.

	Response pattern	Fresh semen	Prefreezed semen	Thawed Semen
Length (µm)	1	8.37±0.05	8.28±0.05^a^	8.17±0.06^a^
	2	8.54±0.05	8.35±0.04^b^	7.96±0.07^c^
	3	8.65±0.07	8.28±0.06^b^	8.23±0.05^b^
Area (µm^2^)	1	32.29±0.36	31.18±0.36^a^	30.90±0.38^a^
	2	33.57±0.38	31.47±0.43^b^	29.70±0.40^c^
	3	33.64±0.36	29.84±0.27^b^	30.61±0.22^b^
p2a	1	1.29±0.01	1.29±0.02^a^	1.29±0.02^a^
	2	1.30±0.01	1.31±0.01^b^	1.28±0.01^c^
	3	1.31±0.01	1.33±0.01^b^	1.31±0.01^a^

Data are mean ± standard error.

*p2a*: perimeter to area (Perimeter^2^/4·π·Area). Different superscripts indicate significant differences (*p*<0.05) among different cryopreservation stages for the same response pattern.

The males characterized as ***“response pattern 2”*** showed a progressive decrease in their sperm head dimensions throughout the cryopreservation process. Changes in sperm head dimensions from fresh to prefreezed and from prefreezed to thawed semen were quite similar, being the change from fresh to thawed semen the highest (Table 1). This did not apply to the p2a (perimeter to area) shape factor because in the transition from fresh to prefreezed stage there was an increase (prefreezing) and then decrease (postthawing) to levels below those observed in fresh semen.

Finally, males belonging to the ***“response pattern 3”*** showed an important decrease of their sperm head dimension from fresh to prefreezed semen, with minimal changes at thawing (Table 1). For these males, total changes on sperm head dimensions took place mainly during the prefreezing stage and these changes were close to 0 from prefreeze to thawing. As for males with response pattern 2, sperm head p2a initially increase on average and then decrease at thawing.

Differences on sperm quality throughout the cryopreservation process between response patterns are presented on Table 2. In fresh and prefreezed semen, the three patterns showed a quite similar sperm quality. Only sperm from males with the *response pattern 3* showed a significant higher viability and percentage of sperm motility. After thawing, sperm quality of males with the *response pattern 2* decreased considerably, being the lowest. Males with the *response pattern 3* showed the best sperm quality, while those males with the *response pattern 1* showed an intermediate sperm quality. When we examined the ratio of change compared to the values in fresh semen (normalized percentages), the same results were observed but with some differences. In this case, the three groups showed a similar decrease in quality during the prefreezing stage, and no differences were observed in the change of the quality of sperm movement among groups. Males showing the *response pattern 2* again showed a greater decline in sperm quality.

**Table 2:** Sperm quality throughout the cryopreservation process for the three patterns of response. Data are mean ± standard error.

		Raw percentages				Normalized percentages*		
	Response pattern	Fresh semen	Prefreezed semen	Thawed Semen (0 h)	Thawed Semen (2 h)	Prefreezed semen	Thawed Semen (0 h)	Thawed Semen (2 h)
Motile sperm (%)	1	80.83±1.11^b^	81.12±0.94^ab^	62.86±2.48^ab^	46.94±2.51^a^	100.62±1.07	77.59±1.18^a^	58.30±2.16^a^
	2	80.17±1.32^b^	78.45±1.84^b^	56.38±3.60^b^	37.07±3.66^b^	97.97±1.95	70.73±1.61^b^	46.74±2.81^b^
	3	84.83±0.94^a^	83.44±1.19^a^	67.59±2.44^a^	50.00±3.21^a^	98.43±1.59	79.87±1.03^a^	59.02±2.85^a^
Quality of sperm	1	3.92±0.08	3.40±0.08	3.72±0.05^ab^	3.28±0.06^a^	87.47±2.38	97.07±2.09	85.91±2.22
	2	3.97±0.11	3.11±0.14	3.46±0.10^b^	3.01±0.11^b^	79.70±3.05	89.11±2.15	77.57±2.35
	3	4.11±0.09	3.42±0.10	3.82±0.05^a^	3.32±0.09^a^	84.41±2.64	94.68±2.66	82.65±2.34
Intact acrosomes (%)	1	90.65±0.65	86.57±0.91	38.14±2.53^b^	28.88±2.38^b^	95.69±0.88	42.50±2.89^b^	32.11±2.67^ab^
	2	89.34±1.24	86.27±1.29	25.70±2.60^c^	21.66±2.62^b^	96.84±1.50	28.79±2.76^c^	24.42±3.06^b^
	3	91.82±0.84	87.34±0.86	49.67±3.04^a^	37.48±3.11^a^	95.17±0.92	53.66±2.14^a^	40.65±3.36^a^
Viability (%)	1	80.16±1.27^ab^	–	38.11±2.04^a^	28.84±1.53^a^	–	47.76±2.56^a^	35.21±1.86^a^
	2	76.98±1.38^b^	–	30.68±2.37^b^	21.71±1.50^b^	–	38.59±2.07^b^	27.31±1.98^b^
	3	82.42±1.20^a^	–	38.95±2.07^a^	31.51±1.81^a^	–	47.00±2.11^a^	37.35±2.26^a^

Different superscripts indicate significant differences (*p*<0.05) among different response patterns for the same stage.

* Normalized percentages regarding to initial semen samples values observed in fresh semen.

We also examined the additive relationships of each male with the other males showing the same response pattern and with those showing different pattern. The within additive relationship coefficients were twice large as that seen between coefficients among groups of response patterns (Table 3). Thus, males who share the same pattern of response were found to be more closely related to each other than with those showing different responses.

**Table 3:** Within (between animals showing the same pattern) and between (among animals showing different pattern) additive relationship coefficients for the three patterns of spermatozoa response during cryopreservation.

	Response pattern		
	1	2	3
Within (%)	8.08±0.24	7.33±0.29	8.49±0.27
Between (%)		4.56±0.23	

Data are mean ± standard error.

## Discussion

The present study has revealed the existence of three distinct patterns of response, based on changes that occur in the dimensions of the sperm head during the cryopreservation process, and how the sperm quality on thawed semen varies depending on these patterns.

The study has been designed to disentangle male effects from other environmental or management effects. Thus, our experimental design was aimed at standardizing the semen collection, as a way to assure that differences in the response to cryopreservation were due to male-specific factors rather than external factors. To achieve this, males have been collected during the breeding season. All males have been maintained in the same conditions. Each male has been collected three times and only those ejaculates showing a good quality were processed and cryopreserved.

Our results support the idea of the existence of an individual component in the response to the cryopreservation process. Many authors have already addressed this issue, and most of them have reported that different individuals exhibit significant different responses to the same freezing protocol [9]–[11]. These authors have considered different sperm characteristics such as sperm morphometry [3], [7], [12], sperm motility and velocity [5], [8], integrity of sperm membranes [4], [17]–[18] or DNA status [6] to evaluate the differences on male response. Here, we have studied the changes that occur in the dimensions of the sperm head during the cryopreservation process.

It is widely accepted that cryopreservation leads to a reduction of sperm head dimensions from fresh to thawed semen [12]–[13], [19]. Different hypothesis have been proposed to explain the reasons why sperm head dimensions decrease after cryopreservation: osmotic changes [20]–[21], possible alterations of some cell compartments [20], [22], damage or loss of the sperm acrosome [23]–[24], overcondesation of sperm nuclear chromatin [25]–[26]. This reduction has been observed in this study as well, but it has not been a general feature of all males. Thus, about half of males (*response pattern 1*) have not changed their sperm head dimensions in a significant way during the cryopreservation. In contrast, the other half of males has undergone changes on their dimensions during cryopreservation. Some of these males (*response pattern 2*) have suffered a progressive decrease in sperm head dimensions, while for the third group of males (*response pattern 3*) changes in sperm head dimensions have mainly occurred just before freezing, during the cooling period. Sperm quality of fresh semen was quite similar for all males regardless of their response pattern, but differed significantly after thawing, with males showing the *response pattern 2* having the lowest quality. The lack of differences of quality in fresh semen would not allow us to characterize the behavior of sperm of a male during cryopreservation based on these parameters [3], [27]–[28]. In contrast, changes in morphometry of sperm heads observed throughout the cryopreservation have proved useful to this purpose.

A number of studies have attempted to address the role of sperm head morphometry on the prediction of freezability [3], [7], [12]–[13], [26]. Although these studies have addressed this goal in different ways, most of them have used related measures of sperm head dimensions on fresh semen with measures of sperm quality at thawing. In general, these studies have reported that males with smaller sperm heads in fresh semen showed better cryoresistance than those with larger heads. Furthermore, some studies have observed that the sperm heads that had a lower rate of change on their dimension, from fresh to thawed semen, had better cryoresistance [12]. The present study provides no information on which sperm parameters are most valuable as predictors of cryoresistance, since we perform a freezing-thawing process to gather information on sperm cryosurvival. Instead, we use this information to *characterize* the sperm cryoresistance of a male. Our work was based on the hypothesis that the response of spermatozoa to the freezing-thawing process could be an inherent feature of males and therefore remain constant. This idea is based on the fact that sperm head morphometry has been showed to be a quite constant feature of males [29]. Here, we propose to characterize the sperm cryoability of a male rather than to predict this ability. Furthermore, we believe that this should be based on the study of patterns of response, rather than on the evaluation of a particular feature at a particular time. Had we focused only in the sperm head dimensions in fresh semen, this study would not have allowed us to identify the role of this feature in predicting cryoresistance. Males that suffered changes on their sperm head dimensions during cryopreservation (*response patterns 2* and *3*) showed significant differences in sperm quality at thawing but were characterized by a similar sperm head morphometry in fresh semen. And those males with *response patterns 1* and *3* showed significant differences in fresh semen but resulted in quite similar quality after thawing. Therefore, it is important to know that the sperm dimensions have changed, but also how these changes occur and at which time of the cryopreservation process.

Cryopreservation involves major changes in sperm cell environment, and their success to survive this process will depend largely on their ability to respond to these changes within the finite time period allowed by the protocol [30]. The responsiveness of spermatozoa to osmotic challenge and their ability to regulate cell volume is a characteristic closely related to cryopreservability [27]. Five distinct processing steps can be recognized in relation with changes in cellular volume [31]: extension and cooling, cryoprotectant addition and packaging, freezing, storage and thawing, each having its special relationship to membrane structure function and cell metabolism. Cooling is known to alter the physical properties of all cell membranes [32]–[33]. The second step demands a large volume change and a rapid recovery [34]–[35]. The addition of cryoprotectant leads to osmotically driven egress of intracellular water, followed by a slower return to the originally volume as the cryoprotectant enters. Freezing alters membrane structure and volume over a very short time period [36]. Extracellular ice crystallization results in very large increases of all other solutes in the remaining liquid and the outward movement of water in response to high concentrations. Finally, thawing requires membrane recovery and cellular expansion.

The changes that occur in sperm volume during cryopreservation and their mechanisms allow us to hypothesize about sperm behavior in term of patterns of response. One of the patterns of response identified in this study has been characterized by maintaining a relatively constant sperm head morphometry throughout the cryopreservation (*response pattern 1*). This would not imply the absence of changes in sperm shape during cryopreservation, but that changes could occur rapidly at each stage. Males showing this response pattern have been characterized by smaller sperm head dimensions in fresh semen and have showed an intermediate sperm quality at thawing. As we pointed out above, many authors have associated this sperm shape with good freezability. In this study, sperm quality at thawing has been higher for those males that have undergone the change on head dimensions mainly during cooling (*response pattern 3*). It is possible that spermatozoa from these males have responded in a better way to the structural changes resulting from the addition of cryoprotectant. The membrane structure function would not have been impaired during cooling allowing the movement of water and cryoprotectant in an efficient manner. Thus, sperm from these males would be less susceptible to damage from freezing and thawing. Finally, we have identified a third pattern of response, in which sperm have changed their morphometry in a progressive way (*response pattern 2*). The males having this response pattern have been characterized by large sperm head dimensions on fresh semen and small dimensions and low quality on thawed semen. Two hypotheses could support the behavior of this sperm: that the sperm membrane structure has been significantly damaged during cooling so the exchange of water and cryoprotectant has occurred in a suboptimal way, or that given the characteristics of their cell membranes, more time is needed to complete the exchange. As a result, spermatozoa would not have responded appropriately to changes in their environment during freezing and thawing, leading to a decrease of quality on thawed semen. We think it would be of utmost importance to further study the cryopreservation process in males that have showed a progressive change on their sperm head dimensions. Confirmation that the membrane structure of spermatozoa would require more time to complete the water and cryoprotectant exchange during cooling would allow the design of specific freezing protocols for each type of male.

This study has allowed us to evaluate whether different ejaculates of a male had the same sperm head dimensions and if these ejaculates showed the same pattern of response throughout the cryopreservation process. Results have showed that, in fresh semen, all sperm head morphometric parameters were quite similar for the different ejaculates of a male, being the coefficients of variation of low magnitude. Also, males have always showed the same pattern of response to cryopreservation for the three ejaculates assessed. The low variability on sperm head dimensions between ejaculates of a male has already been stated for this breed. Thus, Maroto-Morales et al. [29] have reported low CV’s for all the sperm head dimensions. These authors have presented the important role of sperm head morphometry on the characterization of spermatozoa of a male given its low intra-male variability, and have supported the genetic determinism of the sperm morphometry pointed out by other authors [3], [37]. To further explore this idea, we examined the degree of relatedness (obtained from the additive relationship matrix) between males showing the same (*within-*) and different (*between-*) response patterns. Results have showed that additive relationship coefficients for males belonging to the same group were twice larger than those among groups of response patterns. Thus, males who share the same pattern of response were found to be more closely related to each other than with those showing different responses. This result supports the idea of a genetic control of sperm cryoresistance.

In conclusion, the present study shows that sperm head morphometry is a key feature to be included in conservation and breeding programs. The assessment of response patterns suggested here allows us to characterize the sperm cryoresistance of a male. Since this pattern of response has resulted to be a constant feature of a male, this gives greater prominence to the idea of designing specific freezing protocols to each animal.

## Materials and Methods

### Animals

A total of 38 adult males from Manchega dairy sheep breed were used in this study. Males were kept at the Regional Centre of Animal Selection and Reproduction (CERSYRA, Spain) at the same environmental conditions. Age of males ranged from 1.5 to 3 years. All males were maintained in the same conditions of housing and feeding. Males were trained for semen collection by artificial vagina and maintained a regimen of regular collection (twice per week). These semen samples are mainly used for artificial insemination as a part of the breeding program of the Manchega breed.

### Ethics Statement

The study was approved by the “Comité de Ética en Investigación de la Universidad de Castilla-La Mancha”. All animal handling was done following Spanish Animal Protection Regulation RD1201/2005, which conforms to European Union Regulation 2003/65.

### Sperm Collection and Cryopreservation

Semen was collected from males from October to November, coinciding with the breeding season. Each male was collected three times. After collection, sperm quality was evaluated. Traits assessed were: percentage of motile spermatozoa, quality of spermatozoa movement, percentage of spermatozoa with intact acrosomes and percentage of live spermatozoa.

To conduct the sperm quality assessment, 5 µl of fresh semen were diluted on 1500 µl of phosphate-buffered saline (PBS; pH 7.5, 310 mOsm/kg) with 0.5% bovine serum albumin. Sperm motility and quality of movement were evaluated by placing 10 µl of diluted sperm between a pre-warmed slide and a 22 mm×22 mm coverslip and examining it at ×100 magnification under phase contrast optics. The percentage of motile sperm was estimated subjectively with values ranging from 0%, when no motile spermatozoa were observed, and 100%, when all spermatozoa were moving. Quality of sperm movement was also estimated subjectively on a scale of 0–5, where 0 is no motility and 5 is vigorous progressive movement.

Acrosomal integrity after cryopreservation is a well-accepted parameter for estimating sperm function and response to cryopreservation [24], [26], especially after relatively long incubation periods to record changes in plasma membrane sensitively [38]. For the assessment of the percentage of spermatozoa with intact acrosomes, 10 µl of sperm suspension in PBS were diluted with 50 µl of a 2% glutaraldehyde in 0.165 M cacodylate/HCl buffer (pH 7.3), and examined by phase-contrast microscopy. A total of 200 spermatozoa were acquired per ejaculate. Percentages of acrosome integrity were recorded.

Sperm viability was assessed in sperm smears stained with eosin-nigrosin. Thus, 5 µl of sperm suspension with PBS and 10 µl eosin-nigrosin solution were mixed on a glass slide placed on a stage at 37°C and 20 s later the mix was smeared and allowed to air-dry. Smears were examined at ×1000 under bright field and 200 spermatozoa per male were examined to evaluate sperm viability. Live spermatozoa were those excluding eosin (from the eosin-nigrosin stain).

Only those sperm samples with a minimum quality (percentage of motile sperm above 80% and quality of movement above 3.5) were cryopreserved, as a way to assure that all sperm samples showed good quality before freezing. If an ejaculate did not meet the requirements of quality was discarded and the male was recollected again to complete the three semen collections. Cryopreservation was performed as described by García-Alvarez et al. [39]. Briefly, fresh semen was diluted with freezing extender Biladyl® (Minitüb, Tiefenbach, Germany) with 20% egg yolk and an osmolarity balanced at 310 mOsm/kg for the Fraction A (non-glycerol fraction). Semen was diluted to 400×10^6^ spermatozoa/ml with Biladyl®, Fraction A, at 30°C. Diluted semen was cooled to 5°C for 2 h and then was further diluted with the same volume of Biladyl®, Fraction B. Sperm samples were allowed to equilibrate at 5°C for 2 h and packed in 0.25 ml plastic straws. At this point, sperm quality was evaluated again. Finally, they were frozen in a programmable biofreezer (IceCube 14S ver. 1.30; SYLAB Geräte GmbH, Neupurkdersdof, Austria) at –20°C/min to –100°C, and at –10°C/min from –100°C to –140°C. Frozen semen was stored in liquid nitrogen (–196°C) for a minimum period of 6 months before thawing. Thawing was performing by dropping the straws in a water bath with saline serum solution at 37°C for 20 s.

After thawing, sperm quality was assessed as for the two previous stages. The assessment took place just after thawing and after 2 hours of incubation at 37°C. The assessment of sperm viability of thawed semen was performed by evaluating sperm membrane stability by flow cytometry using the combination of YO-PRO-1 and propidium iodide [40].

### Assessment of Sperm Head Morphometry

For each step of the cryopreservation process, sperm head dimensions were assessed objectively by using the morphometric module of the Sperm-Class Analyzer® (SCA) (Microptic, Barcelona, Spain). Sperm morphometry analysis was performed as described by Maroto-Morales et al. [29]. Briefly, microscope slides were prepared by placing 5 µl of sperm diluted in PBS on the clear end of a frosted slide and dragging the drop across the slide. Semen smears were air-dried and stained using the commercial kit Hemacolor™ (Merk, cat. No. 11661, Darmstadt, Germany). Then, stained samples were permanently mounted to the slide with a coverslip and dibutyl phthalate xylene (DPX). A total of 200 images were acquired per ejaculate to ensure a minimum of 125 properly measured sperm heads after improperly measured heads were removed from the analyses.

Morphometry traits assessed were: sperm head length (L, µm), width (W, µm), area (A, µm^2^), perimeter (P, µm), and p2a (perimeter to area; Perimeter^2^/4·π·Area) shape factor. The latter compares the perimeter of an object with its area. The p2a takes a minimum value of 1 when the object is a circle and increases its values for more elongated objects.

### Statistical Analysis

To examine the changes that occur on sperm head dimension throughout the cryopreservation process, the five sperm head morphometry parameters described above were used. However, we focused mainly on three sperm head measures, the sperm head length, sperm head area, and the p2a sperm head factor when displaying these differences. These three parameters have been shown to be sufficient to describe the dimensions of the sperm head in a precise way [29]. Coefficients of variation (CV) among ejaculates were calculated to evaluated the within-male (between ejaculate of the same male) variability.

For the recognition of the different patterns of response, an initial graphical exploration of the sperm population head dimensions throughout the cryopreservation process were performed. Scatter plots of the sperm head area against the sperm head length and p2a were drawn for each male and each stage of the cryopreservation process (see Figures S1 and S2). Three different patterns of response were observed. Males were assigned to one of these patterns based on the following rules: if no significant differences were observed among the stages of the cryopreservation process (fresh, prefrezee and post-thaw) for the 3 sperm head morphometry parameters listed above males were assigned to *response pattern 1*; if sperm morphometry significantly differed for the three stages, males were assigned to *response pattern 2*; and if head morphometry in fresh semen was significant different from the other to stages, being the latter similar, males were assigned to *response pattern 3*. For the particular case in which a male did not meet these requirements for the three morphometric parameters, it was assigned to the closest group. For that, graphical visualization of data was considered as well (see Figures S1 and S2).

Once males were assigned to one of the different pattern of response, multiple comparisons between different stages of the cryopreservation process within each response pattern, and between different responses patterns within each cryopreservation stage were carried out. This analysis allowed us to examine if males showing different patterns were significantly different for all stages or only at a certain stages. The analysis was performed by using a mixed-effects model that account for male variability (random effect). For multiple comparison analyses, Holm’s correction was applied. When comparing semen quality among different group of males throughout the conservation process, we also calculated the standardized percentages defined as the ratio between values at freezing stage compared to values at the initial stage (fresh semen). This allows us to evaluate changes during cryopreservation more accurately.

To examine relatedness between males, additive relationships coefficients were estimated. All males included in this study were from the same generation (contemporaneous). A pedigree consisting of 391,691 individuals of Manchega sheep breed were used to construct the additive genetic relationship matrix, ***A***. Within relationship coefficients were calculated as the average of additive genetic relationship among all males belonging to the same group. Between relationships coefficient was calculated as the average of additive genetic relationship of males of one group with those males belonging to the other two groups.

All statistical were performed using the R (2.15.0) software package (R Development Core Team 2012) [41].

## Supporting Information

Figure S1 **Patterns of response to cryopreservation I.** Relationship between sperm head area and length for the 38 males used in this study throughout the cryopreservation process: fresh semen (red points), pre-freezing (yellow points) and post-thawing (blue points). Numbers within a circle in the lower-right corner of graphs indicate to which pattern each male was assigned.
(TIF)

Figure S2 **Patterns of response to cryopreservation II.** Relationship between sperm head area and p2a relationship for the 38 males used in this study throughout the cryopreservation process: fresh semen (red points), pre-freezing (yellow points) and post-thawing (blue points). Numbers within a circle in the lower-right corner of graphs indicate to which pattern each male was assigned. *p2a*: perimeter to area (Perimeter2/4·π·Area).
(TIF)

## References

[1] Stolovicki E, Braun E (2011) Collective dynamics of gene expression in cell populations. PLoS ONE 6(6): e20530. 21698278 10.1371/journal.pone.0020530 PMC3115940

[2] Benson JD, Woods EJ, Walters EM, Critser JK (2012) The cryobiology of spermatozoa. Theriogenology 78: 1682–1699. 23062722 10.1016/j.theriogenology.2012.06.007

[3] Thurston LM, Watson PF, Mileham AJ, Holt WV (2001) Morphological sperm subpopulations defined by fourier shape descriptors in fresh ejaculates correlate with variation in boar semen quality following cryopreservation. Journal of Andrology 22: 382–394. 11330638

[4] Januskauskas A, Johannisson A, Rodríguez-Martínez H (2003) Subtle membrane changes in cryopreserved bull semen in relation with sperm viability, chromatin structure, and field fertility. Theriogenology 60: 743–758. 12832022 10.1016/s0093-691x(03)00050-5

[5] Martínez-Pastor F, García-Macias V, Alvarez M, Herraez P, Anel L, et al (2005) Sperm subpopulations in Iberian red deer epididymal sperm and their changes through the cryopreservation process. Biol Reprod 72: 316–327. 15385419 10.1095/biolreprod.104.032730

[6] Hernández M, Roca J, Ballester J, Vazquez JM, Martínez EA, et al (2006) Differences in SCSA outcome among boars with different freezability. Int J Androl 29: 583–591. 17121656 10.1111/j.1365-2605.2006.00699.x

[7] Núñez-Martínez I, Morán JM, Peña FJ (2007) Identification of sperm morphometric subpopulations in the canine ejaculate: do they reflect different subpopulations in sperm chromatin integrity? Zygote 15(3): 257–266. 17637107 10.1017/S0967199407004248

[8] Ramón M, Martínez-Pastor F, García-Álvarez O, Maroto-Morales A, Soler AJ, et al (2012) Taking advantage of the use of supervised learning methods for characterization of sperm population structure related with freezability in the Iberian red deer. Theriogenology 77: 1661–1672. 22341709 10.1016/j.theriogenology.2011.12.011

[9] Holt WV (2000) Fundamental aspects of sperm cryobiology: the importance of species and individual differences. Theriogenology 53: 47–58. 10735061 10.1016/s0093-691x(99)00239-3

[10] Thurston LM, Watson PF, Holt WV (2002) Sperm cryopreservation: a genetic explanation for species and individual variation. Cryo Letters 23: 255–262. 12391486

[11] Soler AJ, García AJ, Fernández-Santos MR, Esteso MC, Garde JJ (2003) Effects of thawing procedure on postthawed in vitro viability and in vivo fertility of red deer epididymal spermatozoa cryopreserved at –196°C. J Androl 24: 746–756. 12954668 10.1002/j.1939-4640.2003.tb02737.x

[12] Esteso MC, Fernández-Santos MR, Soler AJ, Montoro V, Quintero-Moreno A, et al (2003) The effects cryopreservation on the morphometric dimensions of Iberian red deer (*Cervus elaphus hispanicus*) Epididymal Sperm Heads. Reprod Dom Anim 41: 241–246. 10.1111/j.1439-0531.2006.00676.x 16689889

[13] Gravance CG, Casey ME, Casey PJ (2009) Pre-freeze bull sperm head morphometry related to post-thaw fertility. Anim Repord Sci 114: 81–88. 10.1016/j.anireprosci.2008.09.014 19013731

[14] Thurston LM, Siggins K, Mileham AJ, Watson PF, Holt WV (2002b) Identification of amplified restriction fragment length poly-morphism markers linked to genes controlling boar sperm viability following cryopreservation. Biol Reprod 66: 545–554. 11870056 10.1095/biolreprod66.3.545

[15] Salamon S, Maxwell WMC (1995) Frozen storage of ram semen I. Processing, freezing, thawing and fertility after cervical insemination. Anim Repord Sci 37: 185–249.

[16] Salamon S, Maxwell WMC (1995) Frozen storage of ram semen II. Causes of low fertility after cervical insemination and methods of improvement. Anim Reprod Sci 38: 1–36.

[17] Martín G, Sabido O, Durand P, Levy R (2004) Cryopreservation induces an apoptosis-like mechanism in bull sperm. Biol Reprod 71(1): 28–37. 14973261 10.1095/biolreprod.103.024281

[18] Sancho S, Casas I, Ekwall H, Saravia F, Rodríguez-Martínez H, et al (2007) Effects of cryopreservation on semen quality and the expression of sperm membrane hexose transporters in the spermatozoa of Iberian pigs. Reproduction 134: 11–121. 10.1530/REP-07-0118 17641093

[19] Peña FJ, Saravia F, García-Herreros M, Núñez-Martínez I, Tapia JA, et al (2005) Identification of sperm morphometric subpopulations in two different portions of the boar ejaculate and its relation to postthaw quality. J Androl 26: 716–723. 16291966 10.2164/jandrol.05030

[20] Gravance CG, Vishwanath R, Pitt C, Garner DL, Casey PJ (1998) Effects of cryopreservation on bull sperm head morphometry. J Androl 19: 704–709. 9876021

[21] Curry MR, Kleinhans FW, Watson PF (2000) Measurement of the water permeability of the membranes of boar, ram, and rabbit spermatozoa using concentrationdependent self-quenching of an entrapped fluorophore. Cryobiol 41: 167–173. 10.1006/cryo.2000.2277 11034795

[22] Arruda RP, Ball BA, Gravance CG, Garcia RP, Liu IKM (2002) Effects of extenders and cryoprotectants on stallion sperm head morphometry. Theriogenology 58: 252–256.

[23] Thomas C, Garner D, DeJarnette J, Marshall C (1997) Fluorometric assessments of acrosomal integrity and viability in cryopreserved bovine spermatozoa. Biol Reprod 56: 991–998. 9096883 10.1095/biolreprod56.4.991

[24] Peña AI, Lugilde LL, Barrio M, Herradon PG, Quintela LA (2003) Effects of Equex from different sources on post-thaw survival, long- evity and intracellular Ca2+ concentration of dog spermatozoa. Theriogenology 59: 1725–1739. 12566147 10.1016/s0093-691x(02)01233-5

[25] Rijsselaere T, Van Soom A, Hoflack G, Maes D, de Kruif A (2004) Automated sperm morphometry and morphology analysis of canine semen by the Hamilton-Thorne analyser. Theriogenology 62: 1292–1306. 15325556 10.1016/j.theriogenology.2004.01.005

[26] Álvarez M, García-Macías V, Martínez-Pastor F, Martínez F, Borragán S, et al (2008) Effects of cryopreservation on head morphometry and its relation with chromatin status in brown bear (*Ursus arctos*) spermatozoa. Theriogenology 70: 1498–1506. 18692226 10.1016/j.theriogenology.2008.06.097

[27] Petrunkina AM, Gröper B, Günzel Apel AR, Töpfer Pedersen E (2004) Functional significance of the cell volume for detecting sperm membrane changes and predicting the freezability in dog semen. Reproduction 128: 829–842. 15579601 10.1530/rep.1.00296

[28] Hidalgo M, Rodríguez I, Dorado JM (2007) The effect of cryopreservation on sperm head morphometry in Florida male goat related to sperm freezability. Anim Reprod Sci 100: 61–72. 16904275 10.1016/j.anireprosci.2006.07.003

[29] Maroto-Morales A, Ramón M, García-Alvarez O, Soler AJ, Esteso MC, et al (2010) Characterization of ram (Ovis aries) sperm head morphometry using the Sperm-Class Analyzer. Theriogenology 73: 437–448. 20018357 10.1016/j.theriogenology.2009.10.003

[30] Mazur P, Cole KW (1989) Roles of unfrozen fraction, salt concentration and changes in cell volumes in the survival of human erythrocytes. Cryobiol 26: 1–29. 10.1016/0011-2240(89)90030-8 2924590

[31] Hammerstedt RH, Graham JK, Nolan JP (1990) Cryopreservation of mammalian sperm: what we ask them to survive. J Androl 11: 73–88. 2179184

[32] Quinn PJ (1989) Principles of membrane stability and phase behaviour under extreme conditions. J Bioenerg Biomemb 21: 3–19. 10.1007/BF00762209 2651426

[33] Peña FJ, Johannisson A, Wallgren M, Rodriguez Martinez H (2004) Antioxidant supplementation of boar spermatozoa from different fractions of the ejaculate improves cryopreservation: changes in sperm membrane lipid architecture. Zygote 12: 117–124. 15460106 10.1017/s096719940400262x

[34] Armitage WJ (1986) Osmotic stress as a factoring the detrimental effect of glycerol on human platelets. Cryobiol 23: 116–125. 10.1016/0011-2240(86)90002-7 3084174

[35] Fiser PS, Fairfull FW (1989) The effect of glycerol-related osmotic changes on post-thaw motility and acrosomal integrity of ram spermatozoa. Cryobiol 26: 64–69. 10.1016/0011-2240(89)90033-3 2924593

[36] Schneider U, Mazur P (1984) Osmotic consequences of cryoprotectant permeability and its relationship to the survival of frozen-thawed embryos. Theriogenology 21: 68–79.

[37] Roldan ER, Cassinello J, Abaigar T, Gomendio M (1998) Inbreeding, fluctuating asymmetry, and ejaculate quality in an endangered ungulate. Proc Biol Sci 265(1392): 243–248. 9493409 10.1098/rspb.1998.0288 PMC1688876

[38] Peña AI, Johannisson A, Linde-Forsberg C (2001) Validation of flow cytometry for assessment of viability and acrosomal integrity of dog spermatozoa and for evaluation of different methods of cryo- preservation. Journal of Reproduction and Fertility 57: 371–376. 11787178

[39] García-Alvarez O, Maroto-Morales A, Martínez-Pastor F, Garde JJ, Ramón M, et al (2009) Sperm characteristics and in vitro fertilization ability of thawed spermatozoa from Black Manchega ram: electroejaculation and postmortem collection. Theriogenology 72: 160–168. 19349069 10.1016/j.theriogenology.2009.02.002

[40] Martínez-Pastor F, Mata-Campuzano M, Alvarez-Rodríguez M, Alvarez M, Anel L, et al (2010) Probes and techniques for sperm evaluation by flow cytometry. Reprod Domest Anim 45(2): 67–78. 20591067 10.1111/j.1439-0531.2010.01622.x

[41] R Development Core Team (2012) R: A language and environment for statistical computing. R Foundation for Statistical Computing, Vienna, Austria. ISBN 3-900051-07-0, URL http://www.R-project.org/

