# Supplementary figures and images for: Sperm Cell Population Dynamics in Ram Semen during the Cryopreservation Process
Source: PLoS One. 2013 Mar 27;8(3):e59189. doi: 10.1371/journal.pone.0059189 (PMC3609831; doi:10.1371/journal.pone.0059189)

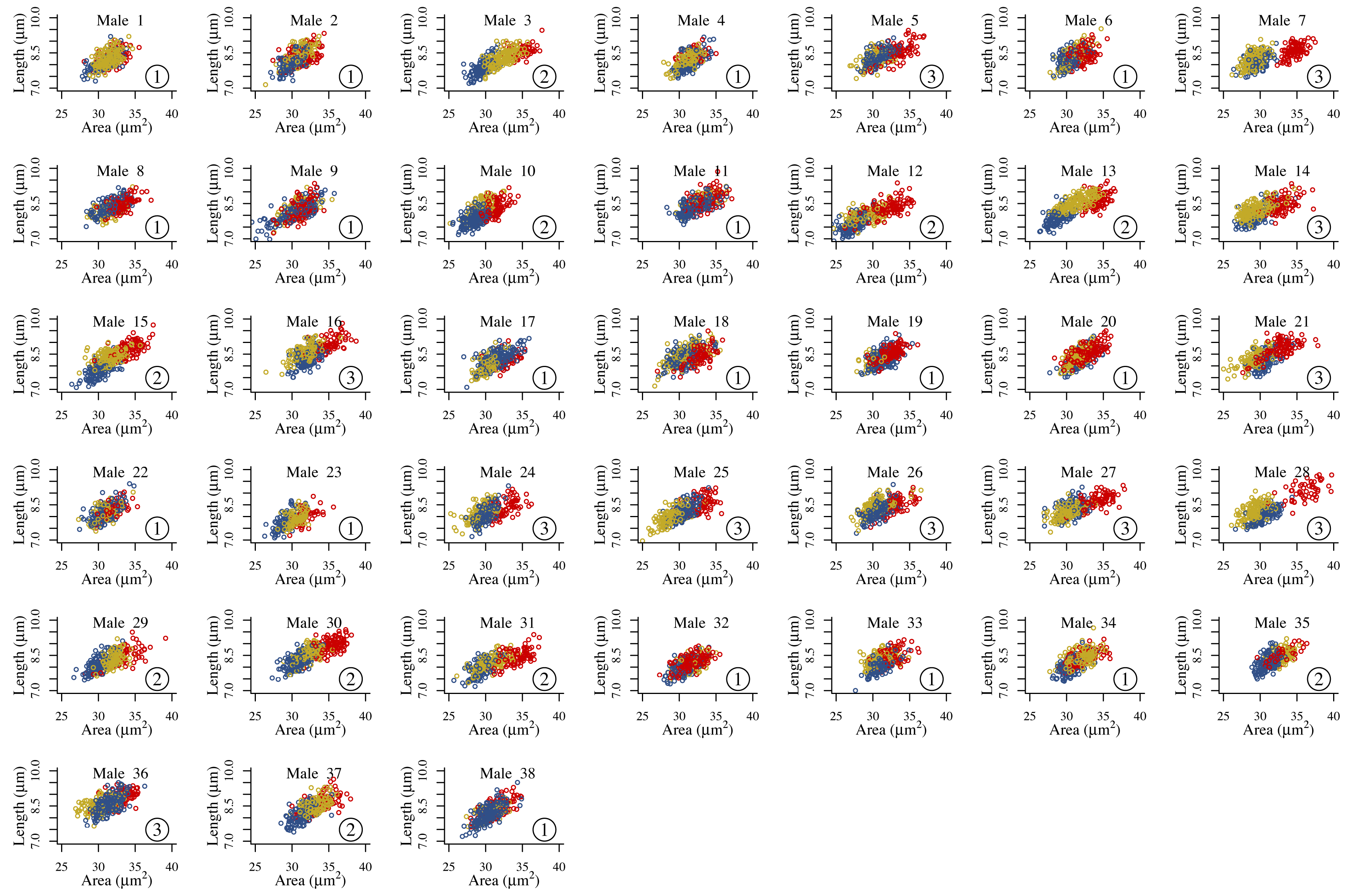

Supplement: Figure S1 — Patterns of response to cryopreservation I. Relationship between sperm head area and length for the 38 males used in this study throughout the cryopreservation process: fresh semen (red points), pre-freezing (yellow points) and post-thawing (blue points). Numbers within a circle in the lower-right corner of graphs indicate to which pattern each male was assigned. (TIF) [file pone.0059189.s001.tif]

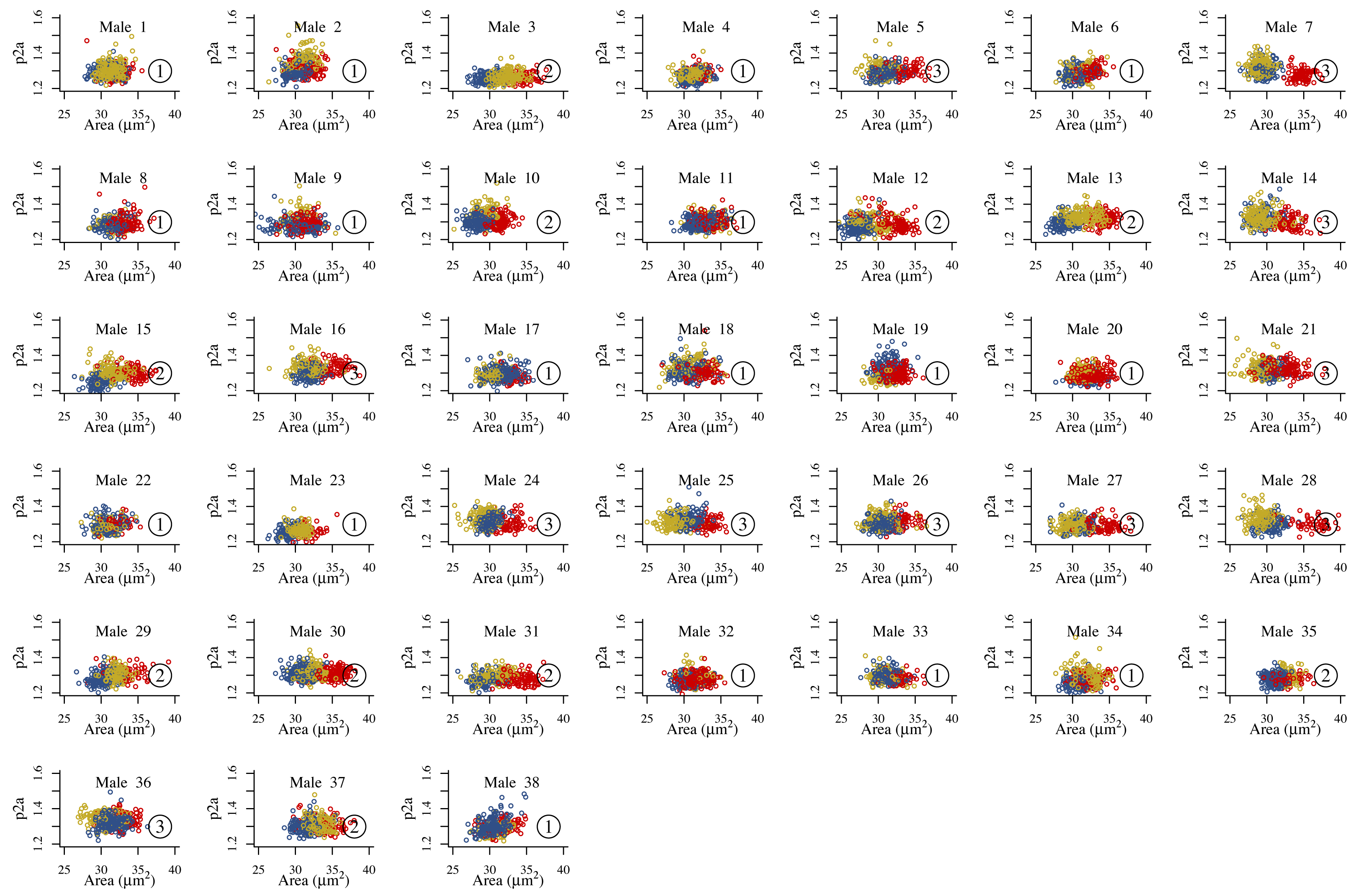

Supplement: Figure S2 — Patterns of response to cryopreservation II. Relationship between sperm head area and p2a relationship for the 38 males used in this study throughout the cryopreservation process: fresh semen (red points), pre-freezing (yellow points) and post-thawing (blue points). Numbers within a circle in the lower-right corner of graphs indicate to which pattern each male was assigned. p2a: perimeter to area (Perimeter2/4·π·Area). (TIF) [file pone.0059189.s002.tif]
